# COP27 climate change conference: urgent action needed for Africa and the world

**DOI:** 10.1136/bmj.o2702

**Published:** 2022-11-09

We introduced errors in the author list of this editorial by Lukoye Atwoli and colleagues (*BMJ* 2022;379:o2459, doi:10.1136/bmj.o2459) during editing. James Kigera and Siaka Sidibé were omitted and as a result the affiliations for many of the authors were also incorrect. The online version has been amended. 

